# Improving the availability of prescription drugs in Lebanon: a critical analysis of alternative policy options

**DOI:** 10.1186/s12961-022-00921-3

**Published:** 2022-10-08

**Authors:** Amena El-Harakeh, Sean J. Haley

**Affiliations:** 1 Department of Community Health and Social Sciences, City University of New York’s Graduate School of Public Health and Health Policy, 55 West 125th Street, New York, NY 10027 United States of America; 2 Department of Health Policy and Management, City University of New York’s Graduate School of Public Health and Health Policy, 55 West 125th Street, New York, NY 10027 United States of America

**Keywords:** Health policy, Health systems, Health systems research, Access to medicines, Generic drugs, Lebanon

## Abstract

The economic downfall in Lebanon and the destruction of the Beirut Port have had a crippling effect on all players in the health sector, including hospitals, healthcare providers, and the pharmaceutical and medical supply industry. The outbreak of COVID-19 has further aggravated the crisis. To address the challenges facing the pharmaceutical industry, Lebanon must create a stable and secure source of prescription drug production. Two alternative approaches are presented to address the crisis: (1) amending the subsidy system and supporting local pharmaceutical production, and (2) promoting the prescription and use of generic drugs. Investing in local production is promising and can lead to establishing trust in the quality of drugs produced locally. These efforts can be complemented by promoting the prescription and use of generic drugs at a later stage, after having had established a well-operating system for local drug production.

## Main text

Pharmaceutical spending in Lebanon accounted for 44% of total healthcare expenditure in 2018 [1], making it the second largest spending component after hospital-based care [2]. Total pharmaceutical costs were nearly US$ 2 billion in 2018, representing 3.3% of the gross domestic product (GDP) [1], compared to an average of 1.6% across countries of the Organisation for Economic Co-operation and Development (OECD) [3]. Lebanon’s per capita pharmaceutical spending is considered one of the highest in the Middle East [4], with over 48% of household annual expenditure spent on medications [5]. This is projected to increase from US$ 281 in 2018 to US$ 438 in 2023 [1]. In addition, more than 40% of the population does not have health insurance, and even with insurance, only 20% of drugs are covered [6].

Prescription drugs represent 83% of pharmaceutical spending [1], with cardiovascular drugs accounting for 20% of the market share, followed by antibiotics (12%), anti-inflammatory and analgesic drugs (9%), and psychotropic medications (5.5%) [7]. The growth in expenditures on pharmaceuticals has corresponded to a considerable increase in the number of pharmacies in Lebanon since 1995, with a 106.8% increase between 2006 (1546 pharmacies) and 2018 (3198 pharmacies) [8, 9]. The ratio of pharmacists has also been on the rise and was estimated at 20.3 to 10,000 population in 2018, compared to a global average ratio of 5.09 to 10,000 population [9].

### Pharmaceutical supply chain

High spending on pharmaceuticals has been coupled with other structural barriers that have reduced the availability of prescription drugs in Lebanon. Despite having 12 local pharmaceutical companies that are certified by WHO for their good manufacturing practices (GMP) and protected by a syndicate of pharmaceutical manufacturers [10, 11], Lebanon’s local pharmaceutical industry is currently operating at only 50% of its capacity [10].

Lebanon imports around 95% of its pharmaceutical products [12]. The sector comprises more than 85 importers [13], and according to the Lebanese Customs Agency, total pharmaceutical imports reached US$ 1.184 billion in 2020 [12]. The central bank has been subsidizing the importation of pharmaceuticals since October 2019 by providing 85% of the foreign currency at an official exchange rate of LL 1507.5 (Lebanese pounds) [14]. This means that importers have to buy the remaining 15% of the foreign currency on the black market, thereby driving up medication prices [14]. Distributors then receive the imported drugs, store them in warehouses and dispense them to pharmacies. Not surprisingly, the Ministry of Public Health has detected increases in smuggling by distributors and pharmacists [15, 16]. Distributors also tend to store large quantities of subsidized drugs, and withhold releasing them to the market until they can sell them later at an unsubsidized price [17]. In addition, over the years, Lebanon has also experienced an inflow of counterfeit drugs [18], and in 2010, counterfeit and falsified medicines were found in 4% of households in Mount Lebanon and in 12% of households in the Bekaa region [19].

Brand-name drugs have the largest share of total pharmaceutical drug sales (49%) and prescription drug sales (61.8%) [7], accounting for 63.2% of local drug production [20]. The production and use of generic prescription drugs remains low, despite evidence of major cost savings due to their low price (20–90% less expensive than brand-name drugs) [6]. A study assessing prescription practices at two community pharmacies in Lebanon found that only 1.8% of prescriptions used generic drugs [21].

In terms of quality, drug analysis and testing are demanded by drug importers and performed by selected laboratories, mostly available in producing countries [5]. Lebanon thus relies on quality control performed outside the country. As for locally produced drugs, two major gaps exist in quality assessment, including (1) the outdated standards of GMP that date back to 1985, and (2) the absence of regular quality assurance audits following the issuance of GMP certificates [7].

### Recent shortages in prescription drugs

Lebanon’s national shortage of prescription drugs peaked during the COVID-19 pandemic [22]. For the past three years, Lebanon has been assailed by crises including economic instability, the COVID-19 pandemic and the destruction of the Beirut Port, all of which have created a catastrophic public health crisis in Lebanon’s fragile healthcare system. As per the World Bank, with an inflation rate of over 230% in January 2022, the economic crisis ranks in the top three most severe crises globally [23]. In a country that relies heavily on importing prescription drugs as well as raw materials for local drug production, the shortage of foreign currency and devaluation of the local one (Lebanese pound) have severely impacted the availability of pharmaceutical products, despite an importation subsidy system financed by the central bank’s foreign reserves. Furthermore, the explosion at the Beirut Port on 4 August 2020 has exacerbated the situation [24]. The prescription drug scarcity has led to citizen stockpiling and to the smuggling of subsidized drugs out of the country via black markets [22, 25].

Lebanon must create a stable and secure source of prescription drug production. Two options that could potentially address the challenge of drug shortages in Lebanon are (1) amending the subsidy system and supporting local pharmaceutical production, and (2) promoting the prescription and use of generic drugs.

### Amending the subsidy system and supporting local pharmaceutical production

Amid the deep financial crisis and with the limited foreign currency reserves, the pharmaceutical sector must reduce its dependence on foreign currency and imported drugs [26]. One policy option is to remove subsidies on imported drugs for which there is an alternative made in Lebanon. In cases where local alternatives are not available, the central bank should restrict subsidies to cheaper alternatives among imported drugs that have similar chemical composition and refrain from subsidizing expensive ones. This is not currently the case, and the central bank subsidizes a wide range of drugs that have similar chemical composition. For instance, it currently subsidizes more than 20 drugs with different trade names for atorvastatin (statin medication), with a price ranging from LL 8977 (US$ 5.95) to LL 42,470 (US$ 28.17) for 30 tablets of 10 mg atorvastatin [25]. Prioritizing subsidies for cheaper imported drugs will help reduce the burden on the central bank and will enhance competition, forcing other importers to lower their prices.

According to the Syndicate of the Pharmaceutical Industries in Lebanon (SPIL), drugs that are manufactured in Lebanon are 30–60% cheaper than their foreign brand-name equivalents, and 15–40% cheaper than their imported generic equivalents [26]. Furthermore, local pharmaceutical companies can cover approximately 68% of the 1500 subsidized imported drugs [27]. As such, given that there is an existing infrastructure for local production, the need will be to increase the scale of operations, not to create a brand-new system. The Lebanese government must entice local industry to increase its market share, with clear short-term targets to address pressing needs of Lebanese patients, and longer-term targets to reduce dependency on imports.

Amending the subsidy system will allow the central bank to channel savings towards financing additional imports of raw materials used in local manufacturing of drugs to support local production. Industry support by the government can also be in the form of offering local pharmaceutical companies financial incentives, including exemption from corporate income tax [28, 29]. This strategy has been employed in other Arab (Bahrain and Kuwait) [30] and developing (India and West Africa) countries and is proven to be effective in supporting local pharmaceutical production [31, 32]. In addition, the government can seek and coordinate financial assistance from developed countries in the form of soft loans (i.e. interest-free or below-market rates of interest) to local pharmaceutical companies [33]. The introduction of this type of financial support has shown to be a catalyst for increasing investment in and the development of the local pharmaceutical industry in Bangladesh and is being proposed to support local pharmaceutical production in Nigeria [34, 35]. These governmental steps should be supported by media coverage to build customer trust in and expand local production [36].

Shortages will not be reduced by increased local production alone. Distribution channels must be held accountable as production increases. Since the Lebanese currency lost more than 90% of its value, [37] drug prices in Lebanon have become the lowest in the region [15]. This has been attributed to increased smuggling by distributors and pharmacists. Current inspections by the Ministry of Public Health are not systematic and are only initiated when there is suspected need. The Ministry must scale up its inspection efforts and perform regular audits that are complemented with disciplinary actions and court referrals for violations. In addition, the Ministry should prioritize the use of locally produced drugs in public clinics and hospitals. This would further expand the market for locally produced drugs.

Another concern pertains to the quality of locally produced pharmaceuticals. There is a lack of trust in the quality of pharmaceuticals in Lebanon, which can be attributed to the absence of a monitoring system for drug quality [36]. Production efforts of local pharmaceutical companies should be overseen and regulated by the Ministry of Public Health to ensure compliance with international GMP. The government should establish a national central laboratory to ensure drug quality pre- and post-registration of drugs [36].

### Promoting the prescription and use of generic drugs

The price of generic drugs in Lebanon is at least 30% less than their comparative brand-name drugs [28]. A law to promote generic drug substitution and the use of generic drugs through the implementation of a unified health prescription (UHP) was adopted in 2015 under Article number 557 [38, 39]. Physicians use a standard prescription form that gives them the ability to select one of two options: (1) marking a check box on the form with a non-substitution (NS) abbreviation, meaning that dispensing a brand-name drug is necessary and generic substitution is highly not recommended, or (2) leaving the corresponding check box blank signalling to the pharmacist the ability to substitute the prescribed medication with a generic alternative [38]. By granting pharmacists the option to propose a generic substitution to the customers, the UHP aims to reduce medication costs by promoting the use of generic drugs [40]. Prior to implementing the UHP, physicians used various prescription forms that do not give them the option to classify drugs as potentially substitutable. Pharmacists would then abide by the physicians’ orders, which are mostly brand-name drugs [41].

Despite efforts by the Ministry of Public Health to support generic drugs, the pharmaceutical sector in Lebanon remains dominated by brand-name drugs, accounting for most of the imported and locally produced drugs [7]. Several studies have shown that there are major challenges hindering the effective implementation of the UHP policy, including resistance from physicians to support generic drugs, and this has been demonstrated by the overuse of the “non-substitutable” drug category on prescription orders [41]. Other challenges include unclear guidance [41], limited efforts by pharmacists to dispense equivalent generic drugs [38], and lack of incentives for pharmacists and providers to promote generic drug use [38, 41, 42]. Pharmacists indicated that their profit margins diminished with the implementation of the UHP policy because of increased sales of “cheaper” generic drugs [43]. Pharmacists in Lebanon are generally reluctant to advocate for “cheaper” generic drugs as they perceive generic drugs to limit their profitability [6].

The success of the UHP policy requires changing the prescription form to make equivalent generics the default selection and requiring orders for non-substitution to be explained. Any change in the standard form will require strong educational campaigns for physicians, pharmacists and the public at large. In addition, the full commitment of both, physicians and pharmacists, and their buy-in must be secured through investing in targeted incentive schemes, including those in which the Ministry of Public Health increases the profit share for pharmacists for selling generic drugs [6]. Professional associations including the Lebanese Order of Physicians and the Lebanese Order of Pharmacists should also play a leading role in ensuring their respective healthcare professionals are educated about the purpose of the policy and are committed to its proper implementation.

At the system level, the Ministry of Public Health, in collaboration with the Lebanese Order of Pharmacists, must develop a comprehensive set of guidelines for pharmacists, including a detailed workflow process to improve the use of the UHP. This will include medication dispensing and generic substitution guidelines for pharmacists. The aim would be to clarify the process and provide a comprehensive and detailed overview of all required information to improve adherence to the UHP policy. The stewardship function of the Ministry of Public Health should also be strengthened through better coordination and oversight of the UHP policy, monitoring and evaluation of implementation including broad population and provider education campaigns, establishing penalties for nonadherence, and introducing tariffs on proprietary medications that have an available generic equivalent.

This approach represents a comprehensive plan to support the use of generic drugs, which are proven to cost at least 30% less than their comparative brand-name drugs in Lebanon [28], while providing the same therapeutic outcomes. In addition, promoting the use of generic drugs is a key component of rational drug prescribing [44]. The main principle of rational drug prescribing is to reduce the reliance on expensive, proprietary drugs when effective cheaper alternatives are available, including generic drugs, so that cost savings can be used to expand the availability of life-saving drugs [45]. Expansion of the UHP policy in Lebanon, coupled with enforcement, will help reduce irrational drug dispensing practices by changing the current prescribing paradigm which defaults to boutique pharmaceuticals to one that prioritizes effectiveness and access [46]. The new system will continue to allow physicians and pharmacists to select between expensive brand-name drugs and generic alternatives. However, improving medication access by expanding the use of effective, less expensive drugs will be prioritized; providers’ selection of elite medications will need to be supported by evidence. The successful implementation of the UHP policy requires the introduction of complementary implementation initiatives that are evidence-based, including stronger regulatory efforts, developing practice guidelines and establishing incentive mechanisms for the healthcare providers [47]. In Sweden, these initiatives, along with other measures such as continuous benchmarking of prescribing patterns, were implemented as part of a generic substitution policy and have led to rational use of drugs [48].

Despite being a multilevel, multicomponent approach to supporting the prescription and use of generic drugs, this second alternative represents a long-term plan that might not provide immediate relief to address the critical shortage of drugs in Lebanon. Mistrust in the efficacy and quality of generic drugs is a common issue in fragile health systems [49]. Over the years, Lebanon has experienced an inflow of counterfeit drugs [18], and in 2010, counterfeit and falsified medicines were found in 4% of households in Mount Lebanon and in 12% of households in the Bekaa region [19]. Substantial, long-term efforts will thus be required to achieve tangible results in terms of changing health providers’ perceptions and practices in relation to generic drug prescription and substitution. Studies have also shown that establishing penalties for nonadherence could be associated with inconsistent implementation efforts [50]. In addition, the current toll of cascading political and economic crises has led to a nearly collapsed health system in Lebanon. This reduces the priority level for implementing training and educational campaigns at a time when patients are struggling to secure prescription drugs.

## Conclusions

### Supporting local pharmaceutical production as an essential step in the way forward

The economic downfall in Lebanon has had a crippling effect on all players in the health sector, including hospitals, healthcare providers, and the pharmaceutical and medical supplies industry. The outbreak of COVID-19 has further aggravated the crisis. To address the challenges facing the pharmaceutical industry, Lebanon needs an emergency plan with immediate measures to tackle the short-term urgency and medium and long-term efforts to sustain pharmaceutical supply. Two alternative approaches are presented to address the crisis: (1) amending the subsidy system and supporting local pharmaceutical production, and (2) promoting the prescription and use of generic drugs.

On the supply side, investing in local production is promising and can lead to establishing trust in the quality of drugs produced locally. Furthermore, local production can improve access to affordable drugs by offering competitive costs compared to international producers [51]. Offering tax reductions or exemptions to local pharmaceutical companies can improve medication affordability and contribute to cost savings [52]. Moreover, increasing the efficiency of the medication distribution system can facilitate access to and promote the rational use of drugs. Inspection efforts by the Ministry of Public Health would need to be bolstered to discourage smuggling by distributors and pharmacists. Evidence shows that the lack of a functioning and sustainable drug supply chain is a major reason for poor access to essential drugs [53].

On the demand side, once generic medications have become more widely available, the standard prescription form must be revised. The current form impedes the rational selection of generics in at least two ways: By offering a simple check box that precludes generic substitution, the form sustains the tiered system without requiring an evidentiary standard. Providers can simply check the box and continue to do what they have always done. In addition, for a generic to be considered, a provider must leave the form blank, which would not seem to promote confidence in generic alternatives or to encourage change in long-standing prescribing practices. Finally, even if the prescriber leaves the form blank, the pharmacist can still fill the prescription with the more expensive medication even when an equivalent is better, continuing the status quo. The prescription form should be changed to make equivalent generics the default selection and require orders for non-substitution to be explained. Any change in the standard form will require strong educational campaigns for physicians, pharmacists and the public at large. Another measure to improve access through expansion of the market for locally produced drugs is prioritizing the use of locally produced drugs in public clinics and hospitals. Once demand has increased, tariffs on proprietary medications that have an available generic equivalent might be introduced to further encourage generic drug selection and production. The entry of lower-cost generic drugs to the market tends to drive up competition and pressures companies that produce brand names to reduce their costs [54]. As such, promoting the development, availability and use of generic drugs is imperative to lower the costs of prescription drugs.

## Data Availability

Not applicable.

## Abbreviations

GDP: Gross domestic product
OECD: Organisation for Economic Co-operation and Development
GMP: Good manufacturing practices
SPIL: Syndicate of the Pharmaceutical Industries in Lebanon
UHP: Unified health prescription
NS: Non-substitution
